# Dead Element Replicating: Degenerate R2 Element Replication and rDNA Genomic Turnover in the *Bacillus rossius* Stick Insect (Insecta: Phasmida)

**DOI:** 10.1371/journal.pone.0121831

**Published:** 2015-03-23

**Authors:** Francesco Martoni, Danna G. Eickbush, Claudia Scavariello, Andrea Luchetti, Barbara Mantovani

**Affiliations:** 1 Dipartimento di Scienze Biologiche, Geologiche e Ambientali, Università di Bologna, Bologna, Italy; 2 Department of Biology, University of Rochester, Rochester, New York, United States of America; University of Muenster, GERMANY

## Abstract

R2 is an extensively investigated non-LTR retrotransposon that specifically inserts into the 28S rRNA gene sequences of a wide range of metazoans, disrupting its functionality. During R2 integration, first strand synthesis can be incomplete so that 5’ end deleted copies are occasionally inserted. While active R2 copies repopulate the locus by retrotransposing, the non-functional truncated elements should frequently be eliminated by molecular drive processes leading to the concerted evolution of the rDNA array(s). Although, multiple R2 lineages have been discovered in the genome of many animals, the rDNA of the stick insect *Bacillus rossius* exhibits a peculiar situation: it harbors both a canonical, functional R2 element (R2Br*^fun^*) as well as a full-length but degenerate element (R2Br*^deg^*). An intensive sequencing survey in the present study reveals that all truncated variants in stick insects are present in multiple copies suggesting they were duplicated by unequal recombination. Sequencing results also demonstrate that all R2Br*^deg^* copies are full-length, i. e. they have no associated 5' end deletions, and functional assays indicate they have lost the active ribozyme necessary for R2 RNA maturation. Although it cannot be completely ruled out, it seems unlikely that the degenerate elements replicate via reverse transcription, exploiting the R2Br*^fun^* element enzymatic machinery, but rather via genomic amplification of inserted 28S by unequal recombination. That inactive copies (both R2Br*^deg^* or 5'-truncated elements) are not eliminated in a short term in stick insects contrasts with findings for the *Drosophila* R2, suggesting a widely different management of rDNA loci and a lower efficiency of the molecular drive while achieving the concerted evolution.

## Introduction

Transposable elements (TEs) are DNA sequence units able to move within the genome. TEs constitute a significant fraction, or even the majority, of some eukaryotic genomes, the percentage reaching 77% in *Rana esculenta* and 85% in *Zea mays* [1]. Their diversity is increasing with new families being continuously discovered, especially as the large number of sequenced genomes is analyzed. The role of transposable elements in evolution is highly debated, but their effects range from beneficial to negative, obviously owing to their impact on host fitness [1, 2, 3].

Class I TEs comprises mobile elements whose movement requires the activity of a reverse transcriptase. A major subclass is represented by non-long terminal repeat (non-LTR) retrotransposons [1]; among them, R2 is one of the most investigated elements and serves as a model for understanding the non-LTR retrotransposition mechanisms. Its structure comprises a single open reading frame (ORF) flanked by two untranslated regions (UTR); the ORF encompasses a central reverse transcriptase (RT) domain which includes RNA binding motifs [4], the DNA-binding motifs at the N-terminus and the endonuclease domain (EN) at the C-terminus. The protein C-terminal end has a cysteine-histidine (zinc finger) motif (CCHC) while the N-terminal domain can contain one (CCHH), two (CCHH + CCHH or CCHC + CCHH), or three (CCHH + CCHC + CCHH) zinc finger motifs ([5] and references therein). R2 has strict sequence specificity for an insertion target site in the 28S rRNA gene (rendering the gene non functional) and it occurs in a wide range of animal taxa, from diploblastic organisms to lower vertebrates [5, 6]. Evolutionarily speaking, R2 belongs to an ancient group of retrotransposons whose members insert specifically into tandem repeats, although a few exceptions have been found [7]. This might represent an adaptive strategy to escape genome purging by limiting damage to a subset of the functional genes among the redundant copies [8, 9].

The R2 mechanism of integration requires a 3’ hydroxyl group at a DNA break to prime reverse transcription (target primed reverse transcription, TPRT [10]). Although the reverse transcriptase occasionally fails to reach the 5’ end of the RNA template, a complete integration event can still take place but the result is a 5’ end truncated copy. The location of the truncation is typically unique; therefore, this length variation at the R2 5’ end can be used to evidence and track the element activity [11, 12].

The R2 RNA template is produced by co-transcription with the rDNA unit followed by self-cleavage. The 5’ end of the R2 elements, in fact, can fold into structures very similar to the self-cleaving ribozymes encoded by the Hepatitis Delta Virus (HDV) [13, 14]. These structures are capable of self-cleavage as demonstrated for the R2 elements of many species with cleavage of the co-transcript occurring upstream of the 28S/R2 5’ junction in many species (for example, in the earwig *Forficula auricularia*) or at the junction of the 28S gene and the 5’ end of the element in some species (for example, in the fruit fly *Drosophila melanogaster*) [15]. This dichotomy in the location of self-cleavage has been correlated with the types of R2 junctions within a species. R2 5’ junctions are uniform for most R2s in which self-cleavage is upstream in the rRNA sequences but they are variable for most R2s in which cleavage is at the R2 5’ end. It has been postulated that the presence of 28S sequences allows the annealing of the first DNA strand synthesized during retrotransposition to the target site and uniformly primes second strand synthesis; in the absence of 28S sequences, priming depends on chance microhomologies between the target site and the first DNA strand [16, 17].

Owing to its location in the array, R2 dynamics is affected by molecular drive which shapes the composition of the rDNA locus. Molecular drive includes a variety of genomic turnover mechanisms (unequal crossing-over, gene conversion, rolling circle replication, etc.) that determines the spread of new units within the same genome (homogenization) and subsequently in the population, through bisexual reproduction (fixation) [18, 19]. This variability pattern is also known as concerted evolution [18].

We recently analyzed R2 in the stick insect species *Bacillus rossius* (R2Br). In addition to a canonical element encoding a 1054 amino acid sequence comprising all known R2 domains and a single ZF motif (CCHH type) at the N-terminal end (named R2Br^*fun*^), a degenerate but closely related (9.2% nucleotide divergence) element has been also isolated, R2Br^*deg*^ [12]. This latter element exhibits 14 frameshift mutations and one stop codon within the open reading frame, and it is at least 5 Myr old as the degenerate element is found in the Italian subspecies *B*. *rossius rossius* and *B*. *rossius redtenbacheri* and also in the North-African *B*. *rossius tripolitanus* A [12, 20].

A population sequencing survey, based on the 3' end of the R2Br element, indicated that all *B*. *r*. *rossius* samples host only R2Br^*fun*^, while *B*. *r*. *redtenbacheri* populations had either only one element variant (R2Br^*deg*^ or R2Br^*fun*^) or both variants in different proportions. Interestingly, no relationships emerged between the presence/absence of a particular R2Br variant and the reproductive strategies (bisexual *vs* parthenogenetic). On the other hand, tracking element activity in these subspecies revealed new R2Br insertions even in the populations showing only R2Br^*deg*^ in the sequencing survey [12]. Moreover, sequence data clearly indicated a mutation pattern of R2Br^*deg*^ consistent with an ongoing replicative activity. We, therefore, suggested that R2Br^*deg*^ could either represent a non-autonomous element that exploits the retrotransposition machinery of an R2Br^*fun*^ not identified in our survey or duplicates along with the host 28S sequences through genomic turn over mechanisms.

In this paper, we delve further into this issue to better understand the mechanisms underlying the R2Br^*deg*^ duplication. We, therefore, analyze in the genomes of three *B*. *rossius* populations three features that have been linked to the retrotransposition activity of functional R2 elements: i) the sequence of the 28S/5' R2 junction of both full-length and truncated elements, ii) the potential to fold the 5’ junction sequences into a HDV-like ribozyme structure, and iii) the ability of the ribozyme to self-cleave the 28S/R2 co-transcript and, thus, to produce a typical mature R2Br RNA.

## Materials and Methods

### Sampling and DNA isolation

Specimens have been collected in areas where specific permission for sampling is not requested, as sampling sites are located in public areas with no restrictive or protection laws enforced. Animals sampled are not endangered or protected species. Individuals of *B*. *r*. *rossius* from Capalbio (GR, Tuscany; roCAP; one male and one female) and Anzio (RM, Lazio; roANZ; two females), and of *B*. *r*. *redtenbacheri* from Patti (ME, Sicily; rePAT; one male and two females) were field collected and frozen at -80°C until molecular analysis. To assure no kinship between the analyzed insects, when possible, specimens were chosen either from different sampling years (Anzio) or from collection sites located at the opposite sides of the same sampling area (Capalbio). Total DNA was extracted from a single stick insect leg or from the whole body with the standard phenol/chloroform protocol. A previous R2Br survey [12] was carried out on the same sampling; moreover, the presently analyzed roCAP female and one female of rePAT are the same specimens used.

### R2 elements isolation, sequencing and analysis

R2 5’ ends were PCR amplified using a primer anchored to the 28S rRNA gene, 64 bp upstream of the R2 insertion site (28SF2: 5'-GTCAAAGTGAAGAAATTCAACGAAG-3'), coupled with two primers anchored inside R2: starting either at base 1917 (RR2Rin: 5'-CCATTCCATTCAATACAGTATCTCC-3') or at base 1424 (R21424r: 5'-AAGCCCAAACAGCAGACGGC-3').

PCR products were ~2000 bp or ~1400 bp long (with 28SF2+RR2Rin or 28SF2+R21424r, respectively) when the full-length element was amplified (i.e., no 5’ end deletions occurred); also shorter amplicons were produced, and these represented truncated variants whose length depended on the extent of the 5’ end deletion.

PCR amplifications were performed in a 50-μl reaction mix using the GoTaq G2 Flexi kit (Promega), following the manufacturer's instructions. Thermal cycling was as follows: initial denaturation step at 94°C for 5 min, 35 cycles of denaturation at 94°C for 30 s, annealing at 48°C for 30 s and extension at 72°C for 2 min, and a final extension at 72°C for 7 min.

Amplicons were run on a 1.5% agarose gel and bands were eluted from the gel using the Wizard SV Gel and PCR Clean-Up System kit (Promega). Fragments were, then, inserted into a pGEM-T Easy vector (Promega) and used to transform *E*. *coli* DH5α cells. Recombinant colonies were PCR-amplified with T7/SP6 primers and sequenced at Macrogen Inc.—Europe Lab. Sequence data are available in GenBank under the acc. nos. KP657751-KP657892.

Sequence alignment with Clustal W algorithm and pairwise genetic divergence (p-distance) were calculated with MEGA v. 6 [21]; Tajima's Ds have been calculated using DnaSP v. 5.1 [22]. The phylogenetic inference has been carried out using MrBayes 3.2.2 [23], setting the GTR model of substitution. The Markov Chain Monte Carlo process was set on two simultaneous tree searches running for 10^6^ generations and tree sampling every 500 generations. Runs’ convergence was assessed through the variance of split frequencies (< 0.01), PSRF ≥ 1.00 and ESS ≥ 200, after a conservative *burn-in* period of 25%.

### DNA templates for T7 co-transcription/cleavage reactions

DNA templates for RNA transcription were generated by PCR amplification of cloned R2 junctions from a specimen of *B*. *r*. *rossius* from Anzio (both functional and degenerate copies) with unincorporated primers and nucleotides removed using a PCR Purification Kit (BioBasics). The specific primers used can be found in the S1 Table. Self-cleavage was assayed as previously described [13]. In short, PCR templates were incubated in transcription buffer with 20 units of T7 RNA Polymerase (Invitrogen) and trace amounts of [α-^32^P]UTP for one hour at 42°C, the reactions stopped on ice by the addition of 4 volumes of 95% formamide, and the denatured RNA products separated on 8M urea, 5% acrylamide gels. After fixing and drying, the gels were exposed to a phosphorimager screen and analyzed using QuantityOne (BioRad).

## Results

### R2 sequences analysis

**Fig 1:**
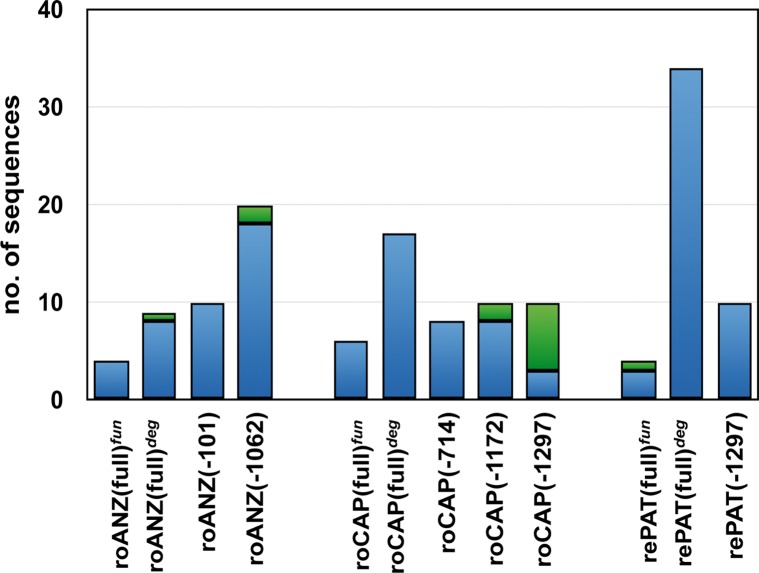
Proportion of unique sequence clones out of the number of sequenced clones. Bins indicate the number of sequenced R2 variants (full-length and truncations) per sample, as listed in Table 1. The blue part of each bin indicates the proportion of unique sequences, i.e. those differing from the others by at least one nucleotide substitution. The green part of each bin represents the proportion of sequences identical to another one.

As a first step to determine how the elements were duplicating in stick insects, the R2 5’ ends were acquired from two specimens of *B*. *r*. *rossius* and one specimen of *B*. *r*. *redtenbacheri* by PCR amplification and cloning. The analysis of 142 clones yielded 74 full-length elements (i. e. without 5’ end deletions) and 68 truncated variants (5’ end deletions ranging from 101 bp to 1297 bp) (Table 1). Over 90% of these clones represents different copies as they had unique sequences, while 13 clones had sequences identical to other clones (Fig. 1).

**Table 1:** Distribution of sequenced R2Br full-length and truncated variants.

5’ end deletion (bp)	roANZ		roCAP		rePAT	
	*fun*	*deg*	*fun*	*deg*	*fun*	*deg*
full-length	4	9	6	17	4	34
-101	10	0				
-714			8	0		
-1062	20	0				
-1172			10	0		
-1297			10	0	10^a^	0

Sequenced variants (indicated by the extent of their 5’ end deletion with respect to the consensus), either functional (*fun*) or degenerated (*deg*), distribution in the three analyzed populations (*B*. *r*. *rossius*; Anzio: roANZ; Capalbio: roCAP. *B*. *r*. *redtenbacheri*; Patti: rePAT).

^a^ Including recombinant elements.

**Fig 2:**
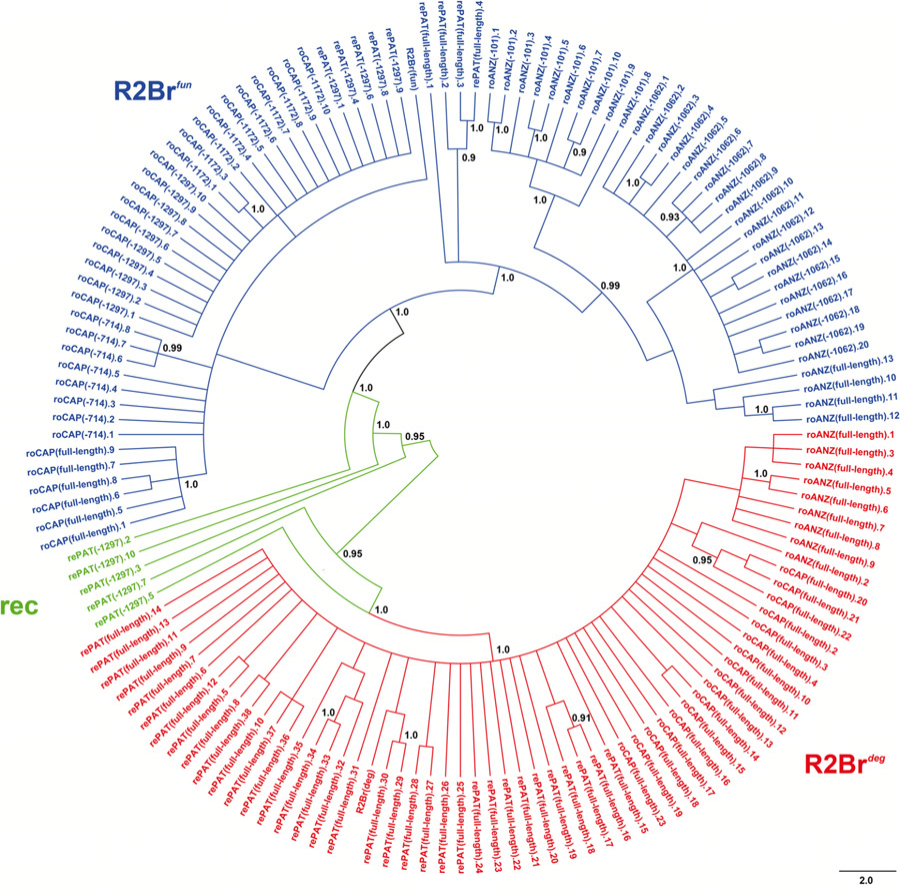
Bayesian phylogeny of the full-length and truncated elements (-ln L = 12418.90). Numbers at nodes indicate posterior probabilities; only values ≥ 0.90 are reported. R2Br^*fun*^ and R2Br^*deg*^ reference sequences are also included as R2Br(fun) and R2Br(deg). The sequences indicated with "rec" are those identified as recombinant between R2Br^*fun*^ and R2Br^*deg*^ (see Fig. 3).

Truncated variants were largely sample-specific, the only exception being the 1297 bp truncation shared between roCAP and rePAT populations (Table 1). The dataset was then analyzed with a phylogenetic method, adding also the reference sequences R2Br^*fun*^ (GenBank acc. no. KJ958674) and R2Br^*deg*^ (acc. no. KJ958675 [12]). The resultant cladogram shows mainly polytomic terminal branches but well-structured clustering at the deepest nodes (Fig. 2). Two main clusters emerge from this analysis: one includes the R2Br^*deg*^ reference element and 34 full-length elements from rePAT, 17 from roCAP and nine from roANZ; the other cluster embodies the R2Br^*fun*^ reference sequence, the remaining full-length elements and most of the truncated variants. The discovery of the degenerate R2 variant in roCAP and roANZ as well as of the functional R2 variant in rePAT was unexpected since in our previous analysis the sequencing of the element's 3’ end did not indicate the presence of these variants in these same populations [12]. It is likely that the different primer pairs used in this study perform better in sampling R2 within the analyzed genomes; moreover, it is to be noted that present samplings involve a 4–5-fold higher number of sequences per analyzed population. While the "degenerate" cluster does not show any sub-structuring, the "functional" cluster exhibits a clear separation between roCAP and roANZ samples with most of the rePAT sequences being intermingled among the other two groups (Fig. 2).

**Fig 3:**
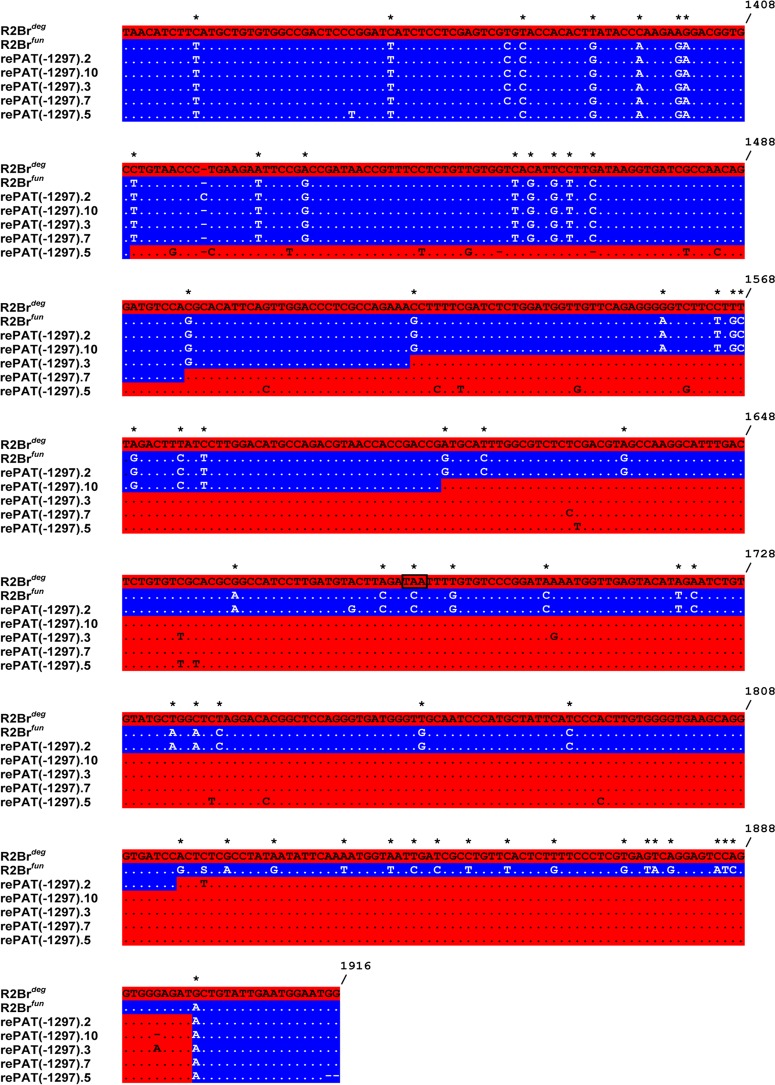
Alignment of recombinant R2Br sequences with the reference sequences, R2Br^*deg*^ and R2Br^*fun*^. Diagnostic sites for distinguishing the two R2Br variants are indicated by asterisks. Different colors shading indicates whether the nucleotide sequence belongs to the R2Br^*deg*^ (red) or R2Br^*fun*^ (blue) element. Numbers at the end of lines refer to nucleotide positions relative to the alignment including the two full-length consensus degenerate and functional sequences. The R2Br^*deg*^ internal stop codon (TAA; [12]) is boxed.

Five rePAT truncated sequences, however, do not clearly fall within either of the two major clusters (Fig. 2). Sequence inspection of diagnostic nucleotides characterizing R2Br^*fun*^ and R2Br^*deg*^ reference sequences revealed that these five sequences are recombinants between the two R2 variants (Fig. 3). More precisely, the five rePAT truncated elements showed R2Br^*fun*^ diagnostic nucleotides at their 5' and 3' ends and, to a different extent, R2Br^*deg*^ diagnostic bases in the internal region. It is likely that these recombinants are the result of gene conversion rather than template switching as the latter would require two jumps from the 5’ end of one transcript to the middle of another transcript; such jumps were not observed during *in vitro* experiments with the R2 protein [24]. Also, the presence of this same truncation in roCAP suggests these recombinant copies were originally derived from the functional variant (Table 1; Fig. 2).

R2Br^*fun*^ sequence variability is quite high with respect to the R2Br^*deg*^ and the 5’ end deleted variants are appreciably more variable than the full-length ones (Table 2). It is to be noted that several unique sequences were detected for all full-length (functional and degenerate) and 5'-truncated element types, the percentage of different sequences ranging from 30% to 100% (Fig. 1). The divergence between the functional and the degenerate R2Br is in line with previous estimates, as well as Tajima’s D values [12]. Quite interestingly, the Tajima’s D calculated only on the full-length R2Br^*fun*^ is not significantly different from zero (Table 2).

**Table 2:** Nucleotide variability and Tajima’s D of sequenced R2Br.

	N	Overall	5' UTR	ORF	Tajima's D^a^
R2Br*^fun^*	82	0.0199	0.0195	0.0198	-1,8064*
Full-length	14	0.0177	0.0263	0.0167	-0,4489^ns^
Truncated	68	0.0208	n.a.^b^	0.0208	-2,2862**
R2Br*^deg^*	60	0.0075	0.005	0.0078	-2,8239***
*fun* vs *deg*	142	0.109	0.153	0.108	

^a^ Probability levels for Tajima’s D statistical significance:

* p < 0.05;

** p < 0.01;

*** p < 0.001;

^ns^ not significant.

^b^ The part of 5’ UTR in the truncated elements dataset is covered only by 10 sequences from the same sample (roANZ) and represents only ~38% of the region: it has been, therefore, not considered.

### The 28S/R2 5’ junctions and the autocatalytic ribozyme

As previously demonstrated for R2 elements in many species, the 5’ end of the R2 RNA is processed from a 28S/R2 co-transcript via an encoded ribozyme [13, 14]. An analysis of the 5’ junctions for the sequenced clones revealed several clues to the putative ribozyme structure(s) for R2Br (Table 3). First, full-length junctions for roANZ, roCAP, and rePAT are uniform suggesting the R2 ribozyme would cleave in the upstream 28S sequences of the co-transcript [15]. Second, while the junctions are also uniform for the degenerate copies, the presence of the non-consensus “A” in the upstream 28S sequences in each junction suggested that a putative ribozyme would have to cleave upstream of this nucleotide in order to regenerate the “A” at the DNA target site. Third, the nucleotide changes in all roCAP full-length junctions also suggested that if it encoded an active ribozyme, it would cleave upstream of these 28S gene changes.

**Table 3:** Sequenced 28S rRNA fragments upstream of the R2 insertion site.

28S sequence	Sample distribution	R2Br variant
GAAGCGCGGGTAAAC*GGCGGGA*GTAACTATGACTCTCTTAAGG	28S consensus	—
GAAGCGCGGGTAAAC*GGCGGGA*GTAACTATGACTCTCTTAA—↓	roANZ, rePAT (full-length); roANZ (-1062)	fun
GAAGCGCGGGTAA-C*ta-tGGA*GTAACTATGACTCTCTTAA—↓	roCAP (full-length)	fun
GAAGCGCGGGTAAAC*GGCGGGA*GTAACTATGACTCTCTT——↓	roANZ (-101); roCAP(-714)	fun
GAAGCGCGGGTAAAC***GGCGGGA***GTAACTATGACTCTC———↓	roCAP (-1297)	fun
GAAGCGCGGGTAAAC*GGCGGGA*GTAACTATGACTCT———-↓	rePAT (-1297)	fun
GAAGC———————————————————↓	roCAP (-1172)	fun
GAAGCGCGGGTAAAC*aGCGGGA*GTAACTATGACTCTCT——-↓	roANZ, roCAP, rePAT (full-length)	deg

The 28S junction sequences detected among the stick insects sampled with the associated R2Br variant (*fun*: functional; *deg*: degenerate) indicated at the far left. Nucleotide substitutions relative to the consensus sequence are in lower-case letters; deletions are denoted with dashes. Arrows mark the positional start of R2 sequences with the species and type (full-length or truncation length) of the element distribution indicated. The portion of the 28S sequence involved in the formation of the ribozyme P1 stem is in italic and underlined.

**Fig 4:**
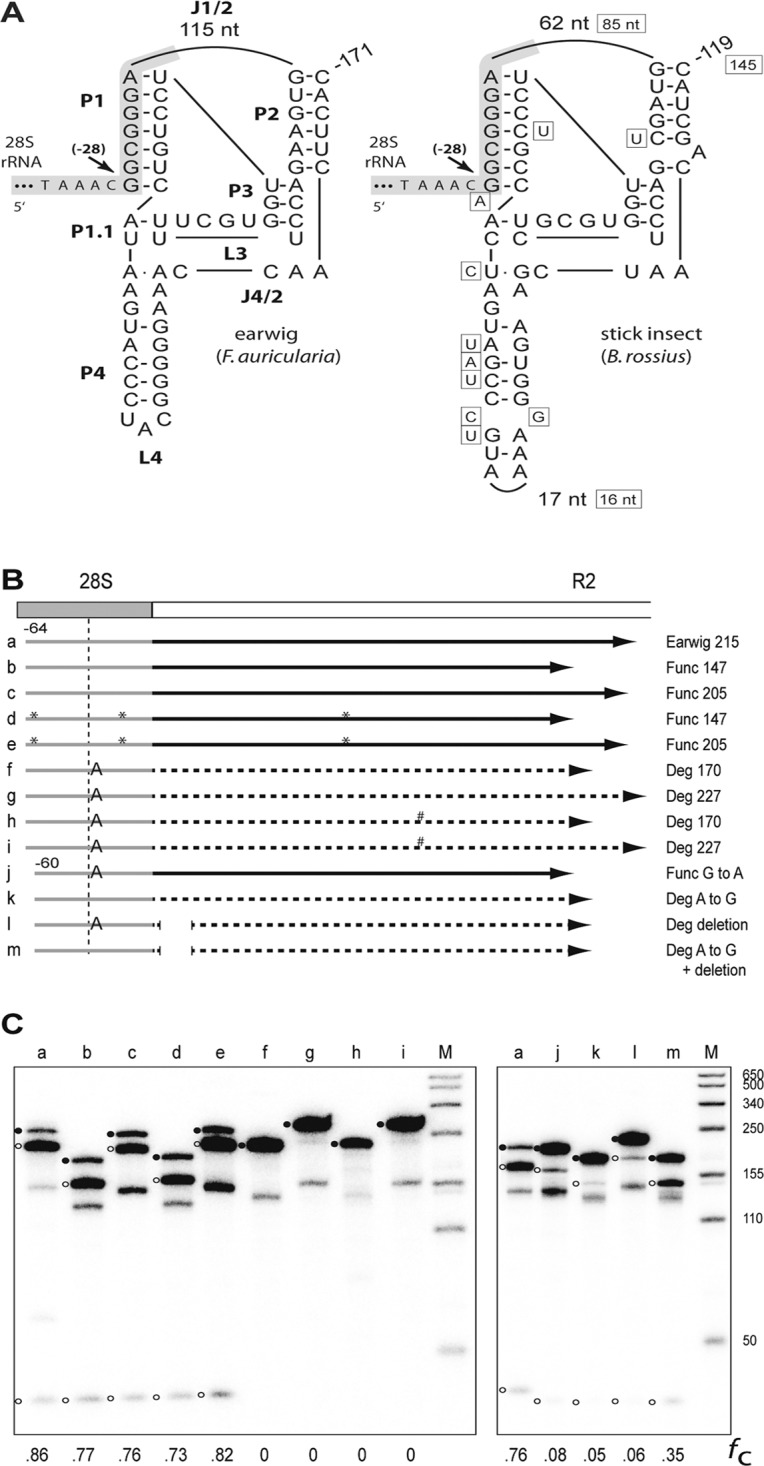
R2Br ribozyme structure and self-cleaving assay. (A) Structure of the R2 ribozyme from the earwig *F*. *auricularia* (left; [14, 15]) and that predicted from the *B*. *rossius* functional element (right) are presented. The predicted RNA secondary structure for the R2Br^*deg*^ element is similar to the latter with nucleotide differences indicated outside the functional element structure (boxed nucleotides). Only the number of R2 nucleotides in the J1/2 loop and the number of nucleotides in the L4 loop of *B*. *rossius* are shown. The 28S gene sequences within and upstream of the ribozymes are shaded gray. Arrows indicate the observed or predicted R2 self-cleavage sites relative to the 3' R2 insertion site. P, base-paired region; L, loop at end of a P region; J, nucleotides joining base-paired regions [13]. (B) Diagram of a generic 28S gene (gray box)/ R2 5' end (white box) junction is shown. Arrows labeled a through m represent the *in vitro* generated RNAs derived from the earwig and stick insect R2s and tested for self-cleavage. RNAs derived from R2Br^*fun*^ are represented by a solid arrow while RNAs corresponding to R2Br^*deg*^ are represented by a dashed arrow. The extent of each RNA relative to the R2 element is indicated on the right. RNAs a-i begin at position-64 relative to the R2 insertion site whereas RNAs j-m begin at position-60. RNAs j-m contain engineered mutations to test their effect on self-cleavage (see text). Nucleotide differences between the two functional copies (*) and between the two degenerate copies (#) are indicated. The vertical dashed line demarcates the predicted cleavage site. (C) 5% denaturing acrylamide gels showing the cleavage products for the RNAs in the co-transcription/self-cleavage assays. The uncleaved RNA (solid circles) and cleavage products (open circles) are indicated. Lanes are labeled with the corresponding letter from panel B. The fraction RNA that self-cleaved is indicated at the bottom. Lane M, RNA length markers with sizes indicated.

A double pseudoknot structure, much like the secondary structures obtained for other insect R2s, could be generated using the sequences from the 5’ junction of the full length R2Br^*fun*^ elements for roANZ and rePAT (Fig. 4A). A very similar secondary structure, albeit with a J1/2 loop which was 23 bp longer, seemed possible for the degenerate variant. Many of the nucleotide differences found in the degenerate element maintained base pairing in the P1, P2, and P4 stems which also suggested that at least at one time it encoded an active ribozyme (Fig. 4A). Consistent with the location of self-cleavage in many species, these structures suggest that an encoded ribozyme would self-cleave at a position 28 nucleotides upstream of the R2 insertion site.

These ribozyme structure predictions suggest that the roCAP full-length junctions (Table 3) are aberrant and would not encode a functional ribozyme. The junction likely originated from an insertion event in which a small portion of the 28S gene sequence at the 5’ end of the RNA was “lost” during cDNA synthesis followed by the addition of non-templated nucleotides at the start of second strand synthesis. The number of full-length elements sequenced is limited; therefore more sequencing and direct experiments are necessary to determine whether this stick insect population contains an active R2. Likewise, the junctions associated with the truncated elements are typical, i. e. they have variable deletions of upstream 28S sequences (2–38 nucleotides, Table 3), and each has at least part of—and roCAP(-1172) the entire—ribozyme secondary structure deleted so none would be able to self-cleave from a potential co-transcript.

### R2Br^fun^ and R2Br^deg^ elements self-cleavage assay

To test for R2Br self-cleavage, templates comprising sequences from two 28S/R2 5’ junctions for the functional element (which differed from each other by two nucleotides in the upstream 28S region and one in the L4 loop) and two degenerate element junctions (which differed from each other by a single substitution in the L4 loop) were generated by PCR amplification of cloned junctions. The T7 generated RNAs tested are diagrammed in Fig. 4B. An earwig R2 RNA was used as a positive control for self-cleavage and as a marker for the location of cleavage. As predicted, the RNAs from the R2Br^*fun*^ element showed self-cleavage which is consistent with a position 28 bp upstream of the R2 insertion site and at a level (average of 77%) comparable to that observed for the earwig ribozyme (86%) (Fig. 4C, lanes a-e). The RNAs derived from the degenerate element, however, had no detectable cleavage (Fig. 4C, lanes f-i).

To corroborate that the degenerate elements were incapable of self-cleavage from a 28S co-transcript, especially given the remarkably similar HDV-like structures envisioned using the functional and degenerate element sequences (Fig. 4A), mutant templates were generated. These new constructs addressed the effect on self-cleavage of specific sequence differences noted between the functional and degenerate variants. First a “G” to “A” substitution was introduced at the base of the P1 stem in the R2Br^*fun*^ element while at the homologous location an “A” to “G” substitution was introduced in the R2Br^*deg*^ element. Self-cleavage decreased almost 10 fold (76% to 8%) for the modified ribozyme from the functional element (Fig. 4C, lanes a and j) while the modified ribozyme associated with the degenerate element now showed a detectable level of self-cleavage (5%, Fig. 4C, lane k). The removal of a 41-nucleotide region which encompasses the 23 bp insertion, as well as multiple sequence differences with the functional J1/2 loop, from the 5’ end of the degenerate element ribozyme also resulted in a comparable increase in self-cleavage (6%, Fig. 4C, lane l). Introducing both the “A” to “G” substitution in the P1 stem and the deletion in the J1/2 loop to the R2Br^*deg*^ element ribozyme resulted in an increase in self-cleavage to 35% (Fig. 4C, lane m). Free energy predictions by an RNA folding program (rna.urmc.rochester.edu/RNAstructureWeb/index.html) for the 5’ junctions of the degenerate RNAs were consistent with these experimental results: the P1 structure predicted for the original R2Br^*deg*^ RNA involved base pairing between the upstream 28S sequences and a region in the J1/2 loop while the prediction for the P1 stem of the doubly modified RNA was as shown in Fig. 4A.

## Discussion

The co-occurrence of multiple R2 elements within the same genome is a well-known situation, with instances of three, or even four, widely divergent R2 elements documented [5, 6, 25, 26]. In *B*. *rossius*, unlike the incidences in *Nasonia vitripennis* or *Tribolium castaneum* for example, this co-presence involves a functional element and its degenerate paralog residing within the same genome. Quite interestingly, the two variants have co-existed for at least 5 Myrs, and the degenerate variant shows the signature of ongoing replication despite the lack of a functional coding region [12]. A previous sequencing survey indicated that R2Br^*fun*^ and R2Br^*deg*^ showed quite different distribution patterns, the former being found as the only resident within the *B*. *r*. *rossius* genome, the latter being present with or without the functional element within different *B*. *r*. *redtenbacheri* populations [12]. The present study based on an intensive survey of the 5’ ends of R2, however, indicates a different scenario as the two variants have been found in both sub-species. The present survey also reveals a peculiar outcome: all sequenced 5'-truncated elements belong to the R2Br^*fun*^ variant and, hence, the R2Br^*deg*^ elements are only full-length. As previously described, a 5' end truncation occurs during an integration event when either the reverse transcriptase falls off before reaching the end of the RNA or the RNA template itself is degraded. The occurrence of 5’ truncations is a characteristic outcome of the non-LTR retrotransposon integration mechanism [27]. However, retrotransposition without evidence of 5’ truncations could still be possible, for example, if the R2Br^*deg*^ element generated RNA which was less prone to degradation.

We, therefore, addressed the structure of the 5’ end of the R2Br^*deg*^ RNA. Typically, mature R2 RNAs are produced by self-cleavage from 28S/R2 co-transcripts through an HDV-like, autocatalytic ribozyme encoded at the 28S/R2 5’ junctions [13, 14]. Our analysis demonstrates that the junction sequences of R2Br are able to form the secondary structure of HDV-like ribozymes and would self-cleave in the 28S gene 28 nucleotides upstream of the insertion site, in line with other R2 ribozymes analyzed in insects [15]. Both R2Br^*fun*^ and R2Br^*deg*^ showed very similar secondary structures, the latter exhibiting point mutations that maintained the ribozyme structure but with a slightly longer J1/2 loop. Self-cleavage assays demonstrate that R2Br^*fun*^ has high levels of activity while R2Br^*deg*^ has no detectable catalytic activity. In particular, the analysis presented here indicates that at least two specific differences can make the R2Br^*deg*^ ribozyme ineffective: the "G" to "A" substitution in the 28S gene that occurs in all sequenced degenerate element junctions, and the additional 23 bp in the R2Br^*deg*^ 5' UTR that appears to interfere with the formation of the P1 stem. Rendering the R2Br^*deg*^ templates more like R2Br^*fun*^ by introducing either an "A" to "G" substitution in the 28S gene or a partial J1/2 loop deletion restores low levels of self-cleavage activity suggesting a stepwise loss of ribozyme functionality. Finally, even if R2Br^*deg*^ is able to self-cleave from the co-transcript at an extremely low level, it is difficult to explain how the “A” nucleotide at the 5’ end of a processed degenerate RNA would be copied into the upstream DNA target sequences (as seen in all sequenced junctions) based on previous models of R2 5’ integration [15].

As previously evidenced [12] and confirmed here by sequence analysis and Tajima's D statistics, R2Br^*deg*^ still replicates. Significantly negative Tajima's D may indicate purifying selection or a sudden explosion of sequence duplications: while the first can be ruled out when dealing with TEs, and particularly with degenerate ones, the latter suggests that R2Br^*deg*^ arose once in the evolution of *Bacillus rossius* and then dramatically increased its copy number. Therefore, there is a mechanism by which R2Br^*deg*^ duplicates despite its inability to encode a protein or to self-cleave and without generating the typical pattern of 5'-truncated copies.

One possibility is that R2Br^*deg*^ behaves as a non-autonomous element, exploiting the enzymatic machinery of the co-existing R2Br^*fun*^, as observed for the R1/R2-derived SIDE elements [28]. Having lost its ability to self-cleave, any non-functional R2 RNA would presumably include at a minimum the sequence from the entire 5’ half of the 28S gene. Even assuming this RNA is stable and escapes mechanisms of rRNA quality control [29], its structure could interfere with the TPRT reaction. For example, a portion of the R2 RNA 5' end is bound by an R2 protein that mediates the second nick on the target DNA during the integration process and performs the second strand synthesis [10, 30]: it is possible that the additional 28S portion at the 5' end inhibits the TPRT process. Finally, the additional sequences at the 5’ end might make it is less likely that degraded transcripts would give rise to the 5’ truncations monitored at the 28S/R2 junction; however, the tendency for the R2 protein to fall off during first strand synthesis would not be effected and truncations generated in this manner should still occur.

Interestingly, 5'-truncated copies, all belonging to the R2Br^*fun*^ variant, show significantly negative Tajima's D and more than one sequence for each 5'-truncated variant occur (Fig. 1): this suggests that once 5'-truncated variants are produced they may further duplicate. On the contrary, Tajima's D calculated on the full-length R2Br^*fun*^ dataset is not different from zero indicating a mutation-drift equilibrium. This is consistent with a stable retrotransposon population where multiple R2 copies duplicate (multiple source model) and others are eliminated through mechanisms of genomic turnover or by drift.

Multiple copies of the same R2 insertion have been scored in *Drosophila* genomes, even if at a frequency lower than that scored in the present analysis, and are thought to be the product of the duplication of the inserted 28S by molecular drive processes [17, 31]. We, therefore, suggest an alternative scenario explaining both the R2Br^*deg*^ and 5'-truncated copies duplication: data presented here are consistent with the hypothesis of duplication through the spread of the original 28S-inserted copy by means of recombination events that are responsible for the concerted evolution of the ribosomal locus (molecular drive, [18, 19]).

This scenario could have major implications for the evolution of TEs targeting a specific site in tandem repeats. As previously reported, R2 belongs to an ancestral clade of non-LTR elements mostly characterized by site-specificity within tandem repeats [8, 9, 32]. The advantages of this strategy can be summarized in four main points: i) insertion in tandem repeats should bring little damage to the host, as uninserted repeats would still be present, ii) tandem repeats such as rDNA will guarantee a transcriptionally active population of insertion sites with which these elements can be co-transcribed, iii) the effect on the host of unequal recombination between retrotransposons should be not different than that between the tandem repeats themselves, while random insertions would lead to harmful ectopic recombination, and iv) molecular drive continuously removes insertions, leading, in the long term, to the survival of only functional (= active) copies. This appears to be the case in many *Drosophila* species where R2 has a replication rate that counteracts the recombinational effects, leading to relatively small R2 populations composed mostly of active elements ([32] and reference therein). The stable maintenance, inheritance and duplication of R2Br^*deg*^ and 5'-truncated copies by 28S molecular drive, though, is deeply inconsistent with the last point and clearly shows that degenerate element survival is possible.

Metazoan genomes have a great excess of rDNA repeats so that, although there are several non-LTR elements and/or at least one DNA element potentially inserting within them [19], there are still enough units to produce the rRNA necessary for the cell to function. R2 elements, for example, vary widely in term of lineage richness and copy number. A single R2 lineage may occupy 10%-45% of the rDNA units in a *Drosophila simulans* genome or 0.5%-5% of the units in the tadpole shrimp *Triops cancriformis* [33, 34]. Moreover, an rDNA array can support up to four R2 lineages, as observed in the sea squirt *Ciona intestinalis*, and up to three lineages have been retrieved in beetles, in the tadpole shrimp *Lepidurus couesii* and in the turtle *Mauremys reevesi* [5, 6, 26, 35]. Although in these instances R2 lineages are all inferred to be functional, based on sequence analysis, it is possible that not all of them are actively retrotransposing. Taking into account the transcription domain model of R2 epigenetic regulation [36], Luchetti and Mantovani [5] suggested that lineages can be silenced while restricted to transcriptionally inactive regions of the rDNA array and/or unleashed when rearranged by unequal crossing over. In this way, also thanks to molecular drive's homogenizing forces, silenced lineages could be maintained over evolutionary time. Based on the empirical data gathered in *Drosophila*, Zhou et al. [31] simulated a population model to explain the interplay between rDNA molecular drive and R2 activity in light of the transcription domain model. They showed that i) the transcriptionally active rDNA domain can be established at each generation in region(s) with no R2 occurrence, ii) that R2 is active only when there is no choice but to transcribe one or more R2-interrupted rDNA unit(s) (e.g. after a contraction of the rDNA units copy number) and iii) that recombination occurs mainly in the transcriptionally active domains. This latter observation would explain why they observed very few R2 insertions duplicated by crossing over.

We have no estimates of R2Br occupancy within the stick insect genome (i.e. the percentage of 28S genes interrupted by an R2Br insertion), but previous inheritance studies highlighted a considerable plasticity of the insertion profile with new insertions and eliminations detectable even in a single generation [12]. Beside active retrotransposition, this speaks in favor of a remarkable rDNA array turnover and indicates that, based on the Zhou et al. [26] model and considering the R2Br^*fun*^ Tajima's D indicating a duplication/elimination equilibrium, one or more copies of R2Br^*fun*^ are currently within the rDNA transcriptionally active domain. On the other hand, the high rate of duplication of 5'-truncated and R2Br^*deg*^ elements observed here clearly contrasts with the Zhou et al. [31] model.

The different behavior of the rDNA/R2 relationship in stick insects with respect to fruit flies can be explained by three, non-mutually exclusive mechanisms. First, in stick insects, recombination is not restricted to the transcriptionally active domain of an rDNA array but may evenly occur throughout the array. Zhou et al. [31] demonstrated that this would lead to duplications of the same insertion but would decrease the number of different R2 insertions. In this view, it is also to be noted that rDNA loci are differentially distributed in the genomes of fruit flies and stick insects, *Bacillus* stick insects having multiple rDNA loci mostly located on autosomes [37, 38] while *D*. *simulans* showing a single rDNA locus on the X chromosome. Therefore, it is possible that, in stick insects, different R2 variants can be located and/or present in different proportions on different rDNA arrays. Where transcription is never or seldom active, but concerted evolution still takes place [31], recombination would occur and in this case the copy number of the retrotranspositionally inactive R2Br^*deg*^ or 5'-deleted R2Br^*fun*^ copies will increase.

Second, functional, 5'-truncated and degenerate elements are all within a transcriptionally active rDNA array because either the stick insect rDNA transcription machinery doesn’t have the ability to select a relatively small R2-free region or there is not an insert-free region for the cell to select. This would place the control of R2 activity and proliferation on the equilibrium between transposition and elimination of active copies by molecular drive, as also data on R2Br^*fun*^ already suggested, and eventually on selective pressures against exceedingly active lineages.

Third, mechanisms of genomic turnover, and therefore concerted evolution, are slower in *B*. *rossius* than in *Drosophila*. In this case, R2-inserted 28S would be less efficiently eliminated in the short term and, on average, persist longer in stick insects. Modeling R2 insertion inheritance in *B*. *rossius* bisexual populations, we showed that the elimination of R2-inserted 28S is mainly driven by selection rather than recombination, in line with a possible low efficiency of genomic turnover mechanisms [12].

Whatever the mechanisms regulating their spread, the present condition of R2Br^*fun*^ 5'-truncated copies and R2Br^*deg*^ allows them to effectively avoid elimination from the array(s). It appears, therefore, that inserting specifically into a tandem repeat array allows dead R2 (and similar TEs) copies to survive and expand their population even if the coding capacity and the possibility to exploit the enzymatic machinery of functional elements have been lost. Taking into account that R2Br^*deg*^ has been maintained for at least 5 Myr, such strategy appears successful. It would be interesting to speculate about how long a dead element can survive in this way and what could be the consequence of their presence within the genome. Many parameters should be calculated to answer these questions (for example, the element occupancy, the rDNA recombination frequency and the number of rDNA loci) but it is likely that a dead element could survive until its population experiences severe contraction, making it more vulnerable to drift. An interesting point of the coexistence of multiple R2 lineages is that they may recombine, potentially generating further lineages [26]. Here we showed that R2Br^*fun*^ elements can recombine with degenerate ones; the gene conversion detected here involved a fragment carrying the stop codon identified in the R2Br^*deg*^ [12] integrating within a functional copy. Although the functional elements that underwent recombination are 5'-truncated, thus unable to retrotranspose, this finding suggests that the possible recombination between R2Br^*fun*^ and R2Br^*deg*^ may inactivate active copies rather than produce new lineages. This may be part of a possible trade-off in maintaining a number of degenerate element copies, and it will be an intriguing issue to better investigate in further studies.

## Supporting Information

S1 Table List of the primers used for obtaining DNA templates for T7 co-transcription/cleavage reactions. Primers have been utilized to generate: i) the original templates (OT), ii) the “G” to “A” mutation in the functional element ribozyme (G>A^fun^), iii) the “A” to “G” mutation in the degenerate element ribozyme (A>G^deg^), iv) the partial J1/2 loop deletion in the degenerate element ribozyme (J1/2 ^loop del^), v) the partial J1/2 loop deletion plus “A to G” double mutation (J1/2 ^loop del^+A>G^deg^). For J1/2 ^loop del^ and J1/2 ^loop del^+A>G^deg^ final products, a nested PCR was performed involving first the *BrLoopDel+BrDeg_227REV* pair, then *T7/28S(G to A)*+*BrDeg_170REV* and *T7/28S(A to G)*+*BrDeg_170REV*, respectively.
(DOCX)
