# Supplementary material for: Dead Element Replicating: Degenerate R2 Element Replication and rDNA Genomic Turnover in the Bacillus rossius Stick Insect (Insecta: Phasmida)
Source: PLoS One. 2015 Mar 23;10(3):e0121831. doi: 10.1371/journal.pone.0121831 (PMC4370867; doi:10.1371/journal.pone.0121831)
Supplement: S1 Table — Primers have been utilized to generate: i) the original templates (OT), ii) the “G” to “A” mutation in the functional element ribozyme (G>Afun), iii) the “A” to “G” mutation in the degenerate element ribozyme (A>Gdeg), iv) the partial J1/2 loop deletion in the degenerate element ribozyme (J1/2 loop del), v) the partial J1/2 loop deletion plus “A to G” double mutation (J1/2 loop del+A>Gdeg). For J1/2 loop del and J1/2 loop del+A>Gdeg final products, a nested PCR was performed involving first the BrLoopDel+BrDeg_227REV pair, then T7/28S(G to A)+BrDeg_170REV and T7/28S(A to G)+BrDeg_170REV, respectively. (DOCX) [file pone.0121831.s001.docx]

**Table S1.** List of the primers used for obtaining DNA templates for T7 co-transcription/cleavage reactions. Primers have been utilized to generate: i) the original templates (OT), ii) the “G” to “A” mutation in the functional element ribozyme (G>A^fun^), iii) the “A” to “G” mutation in the degenerate element ribozyme (A>G^deg^), iv) the partial J1/2 loop deletion in the degenerate element ribozyme (J1/2 ^loop del^), v) the partial J1/2 loop deletion plus “A to G” double mutation (J1/2 ^loop del^+A>G^deg^). For J1/2 ^loop del^ and J1/2 ^loop del^+A>G^deg^ final products, a nested PCR was performed involving first the *BrLoopDel+BrDeg_227REV* pair, then *T7/28S(G to A)*+*BrDeg_170REV* and *T7/28S(A to G)*+*BrDeg_170REV*, respectively.

| **Amplicon** | **Primer names** | **Upstream primer (5' > 3')** | **Downstream primer (5' > 3')** |
| --- | --- | --- | --- |
| OT | *T7/28S(-63G)* | TAATACGACTCACTATAGGGgCAAAGTGAAGAAATTCAACGAAGCG |  |
| OT | *T7/28S(-63T)* | TAATACGACTCACTATAGGGtCAAAGTGAAGAAATTCAACGAAGCG |  |
| OT | *Br^fun^_147REV* |  | CCAGCCCCGGGTACCAGC |
| OT | *Br^fun^_205REV* |  | CCATCCGAGGCTTCTTTTTGTAAGAAC |
| OT | *Br^deg^_170REV* |  | CTGGCCCCGGGTACCATC |
| OT | *Br^deg^_227REV* |  | CATCCGAGGCTTCATTTTGCGAAATC |
| G>A^fun^ | *T7/28S(G to A)* | TAATACGACTCACTATAGGGAAGTGAAGAAATTCAACGAAGCGCGGGTAAACAGCGGGAG |  |
| G>A^fun^ | *Br^fun^_147REV* |  | CCAGCCCCGGGTACCAGC |
| A>G^deg^ | *T7/28S(A to G)* | TAATACGACTCACTATAGGGAAGTGAAGAAATTCAACGAAGCGCGGGTAAACGGCGGGAG |  |
| A>G^deg^ | *Br^deg^_170REV* |  | CTGGCCCCGGGTACCATC |
| J1/2 ^loop del^ | *BrLoopDel* | GCGCGGGTAAACAGCGGGAGTAACTATGACTCTCTTGGAGGCTGAGGAGATCG |  |
| J1/2 ^loop del^ | *Br^deg^_227REV* |  | CATCCGAGGCTTCATTTTGCGAAATC |
| J1/2 ^loop del^ | *T7/28S(G to A)* | TAATACGACTCACTATAGGGAAGTGAAGAAATTCAACGAAGCGCGGGTAAACAGCGGGAG |  |
| J1/2 ^loop del^ | *Br^deg^_170REV* |  | CTGGCCCCGGGTACCATC |
| J1/2 ^loop del^+A>G^deg^ | *BrLoopDel* | GCGCGGGTAAACAGCGGGAGTAACTATGACTCTCTTGGAGGCTGAGGAGATCG |  |
| J1/2 ^loop del^+A>G^deg^ | *Br^deg^_227REV* |  | CATCCGAGGCTTCATTTTGCGAAATC |
| J1/2 ^loop del^+A>G^deg^ | *T7/28S(A to G)* | TAATACGACTCACTATAGGGAAGTGAAGAAATTCAACGAAGCGCGGGTAAACGGCGGGAG |  |
| J1/2 ^loop del^+A>G^deg^ | *Br^deg^_170REV* |  | CTGGCCCCGGGTACCATC |
